# Negative Parenting Attitudes and Adolescent Suicidal Ideation: The Moderated Mediating Role of Academic Helplessness and Organized Youth Activity Participation

**DOI:** 10.3390/bs16091565

**Published:** 2026-09-03

**Authors:** Youngchae Shin, Sung-Man Bae

**Affiliations:** 1 Department of Psychology, Graduate School, Dankook University, Cheonan 31116, Republic of Korea; etre_syc@dankook.ac.kr; 2 Department of Psychology, College of Health Science, Dankook University, 119 Dandae-ro, Cheonan 31116, Republic of Korea

**Keywords:** negative parenting attitudes, suicidal ideation, academic helplessness, organized youth activity participation, adolescents, moderated mediation

## Abstract

This study examined the conditional indirect association between negative parenting attitudes and suicidal ideation via the mediation of academic helplessness, and investigated whether organized youth activity participation moderated this indirect association. Data were drawn from Wave 6 (2023) of the Korean Children and Youth Panel Survey (KCYPS 2018), with a final sample of 2224 adolescents aged 17–18 years. Gender and subjective health perception were included as control variables. PROCESS Macro Models 4 and 7 were applied to test the indirect effect and analyze the moderated mediation effect, respectively. Negative parenting attitudes were significantly associated with suicidal ideation, and the partial mediating role of academic helplessness was also significant. Organized youth activity participation significantly moderated the association between negative parenting attitudes and academic helplessness, and the indirect effect of negative parenting attitudes on suicidal ideation via academic helplessness varied significantly as a function of the level of organized youth activity participation. These findings suggest that organized youth activities may function as a possible effect modifier, consistent with the principles of behavioral activation theory, attenuating the indirect association from negative parenting attitudes to suicidal ideation via academic helplessness. Practical implications of these findings for research and practice concerning adolescent suicidal ideation are discussed.

## 1. Introduction

The suicide rate in South Korea is a severe public health concern, consistently ranking highest among Organization for Economic Co-operation and Development (OECD) member countries since 2003. Notably, suicide is the leading cause of mortality among Korean adolescents, with a rate of 11.7 per 100,000 individuals. This figure is significantly higher than the second leading cause of death—unintentional injuries—which accounts for 2.3 per 100,000 deaths (Korea Foundation for Suicide Prevention, 2025). Adolescence is a developmental transition and preparatory phase for adulthood, characterized by profound physical, emotional, and social changes. As self-regulatory capacities are not fully developed during this period, adolescents are significantly more vulnerable to stress than adults, demonstrating a higher propensity for emotional and behavioral problems (K. J. Lee et al., 2023).

Suicidal ideation is defined as thoughts of engaging in behavior with the intent to end one’s life (Nock et al., 2008). It is conceptualized as a broad construct encompassing both passive suicidal ideation, characterized by a general wish to die, and active suicidal ideation, which involves specific motivations, intentions, and plans (Wastler et al., 2023). Recent evidence indicates that the experience of suicidal ideation does not inevitably culminate in suicidal behavior (Silverman et al., 2007), and the precise mechanisms underlying the transition from suicidal thoughts to actions remain poorly understood (Klonsky et al., 2018). Nevertheless, suicidal ideation is established as a significant risk factor for suicide attempts (Franklin et al., 2017). Adolescents are particularly vulnerable to suicidal thoughts, as this developmental stage involves a critical physical, psychological, and social transition into adulthood (K. J. Lee et al., 2023). Therefore, rigorous investigation of suicidal ideation among the adolescent population is of paramount importance.

Previous research categorizes the factors associated with adolescent suicidal ideation into psychological, domestic, and socio-cultural domains (Tintori et al., 2023; Prades-Caballero et al., 2025). Among these, familial factors show a continuous and fundamental association with adolescent development; a meta-analysis synthesizing a decade of South Korean studies on adolescent suicidal ideation also indicated a strong correlation with suicidality (K. J. Lee et al., 2023). Throughout the developmental trajectory of adolescence, parenting attitudes are profoundly associated with the personality, self-esteem, value systems, and emotional well-being of offspring (Krauss & Orth, 2024). Furthermore, adolescents who perceive their parents as exhibiting inconsistent, excessively intrusive, coercive, or rejecting attitudes report a significantly higher prevalence of suicidal ideation (Macalli et al., 2018; Sachs et al., 2024).

Adolescents spend most of their time at school, rendering academic achievement a primary developmental task. In South Korea—which places profound emphasis on university education—academic issues are recognized as directly tied to high school students’ sense of self-worth (Jang & Jeong, 2023). According to the 2022 Education Suffering Index, 25.9% of students reported having contemplated self-harm or suicide due to anxiety and depression stemming from their academic performance (The World Without Worries About Shadow Education, 2022). Given an average high school class size of 25 students, this prevalence equates to approximately six to seven students per classroom, indicating that academic-related emotional distress significantly affects adolescent suicidal ideation.

During the high school period preceding university admission, the sense of frustration stemming from minor failures may be perceived more acutely. Repeated exposure to such frustration renders students vulnerable to helplessness, driven by the belief that outcomes are beyond their control (Abramson et al., 1978). Academic helplessness is defined as a state in which adolescents, following repeated failures in academic settings, perceive that a desired level of academic achievement is unattainable regardless of their ability or effort. Consequently, they cease their attempts to affect change, resulting in difficulties with normal academic performance (Bak et al., 2015). Academic helplessness has also been closely associated with negative parenting attitudes (Han & Ha, 2021). When negative parenting is accompanied by repeated academic failures, adolescents tend to demonstrate extremely low levels of self-efficacy. This is associated with a diminished sense of perceived control, thereby precipitating the experience of academic helplessness (Han & Ha, 2021). The sense of loss of control formed through repeated failures may lead to a loss of hope for the future (Abramson et al., 1978), which in turn may serve as a key factor precipitating suicidal ideation. A study utilizing machine learning to analyze the longitudinal trajectories of adolescent suicidal ideation identified academic helplessness as a significant predictor distinguishing high-risk groups, independently of existing emotional variables (T. Lee & Kim, 2023).

Based on the previous studies, negative parenting attitudes may be associated with suicidal ideation through the mediation of academic helplessness. Therefore, in order to reduce the risk of adolescent suicide, it is important to understand the possible effect modifiers that can buffer the pathological association between negative parenting attitudes and suicidal ideation. Previous studies have primarily focused on emotional and psychological characteristics such as self-esteem and social support (Marraccini & Brier, 2017; Vancampfort et al., 2018); consequently, the exploration of behavioral protective factors that can be practically intervened upon in adolescents’ daily lives remains insufficient. In this context, attention must be paid to organized youth activities (Mahoney et al., 2006; Hansen et al., 2003), which are distinct from simple leisure activities, as they are characterized by structured approaches and clear goals and rules, offered in diverse forms, with adolescents participating voluntarily (Eccles et al., 2003). Organized youth activities in Korea are grounded in the Youth Activity Promotion Act and encompass a broad spectrum of programs such as volunteer work, arts, physical activities, career exploration, and exchange programs (Ministry of Government Legislation, 2016). These activities go beyond simple leisure, providing opportunities to promote autonomy, a sense of belonging, and positive reinforcement (Hansen et al., 2003). They have the advantage of high accessibility, as they are facilitated by schools or local communities, and serve as an effective mechanism for stress reduction (Oh & Ryu, 2017). Furthermore, they contribute to the formation of adolescents’ self-concepts and enhance their quality of life, encouraging proactive engagement in improving their own environments (N. J. Kim & Im, 2012).

The voluntary participation and structured nature of organized youth activities are consistent with the principles of behavioral activation theory, which emphasizes positive reinforcement experiences. According to behavioral activation theory, depression can be understood as a product of diminished positive reinforcement experiences and heightened avoidance behaviors. Individuals resort to avoidance behaviors to temporarily escape psychological discomfort; however, such behaviors may further compound emotional and behavioral difficulties by precluding opportunities to experience positive reinforcement (Ferster, 1974; Lewinsohn, 1974). Conversely, increasing engagement in meaningful and value-congruent activities enhances exposure to positive reinforcement and attenuates negative emotional states (Lejuez et al., 2001). Organized youth activities, by affording experiences of social interaction, skill acquisition, belonging, and autonomy, may foster competence and social connectedness, and have been associated with lower levels of suicidal ideation (Mahoney et al., 2002; Armstrong & Manion, 2015). Prior research has indicated that participation in organized youth activities enhances interpersonal skills through interaction with adult figures such as instructors and teachers, exerting a positive association with parent–child relationships; furthermore, a higher frequency of activity participation has been associated with lower levels of academic helplessness (E. T. Lee & Cho, 2025). Crandall et al. (2024) found that positive activity experiences were associated with lower levels of learned helplessness, even in the presence of adverse childhood experiences (ACEs); structured after-school activity participation has also been associated with adolescent suicidal ideation. Collectively, these findings suggest that organized youth activity participation (OYAP) may moderate the magnitude of the pathway through which adverse parenting environments are associated with academic helplessness. Although prior studies have investigated these variables in isolation, an integrated model examining how OYAP moderates the indirect pathway from perceived negative parenting attitudes to suicidal ideation via academic helplessness remains largely unaddressed. Accordingly, the current study seeks to explore a comprehensive mechanism underlying suicidal ideation by integrating these variables into a single model. Examining this integrated pathway holds empirical significance, particularly for South Korean high school seniors who experience severe academic pressure in preparation for university entrance examinations. As the present study is exploratory in orientation and relies on cross-sectional data, the consideration of alternative models, including the possibility of reverse or reciprocal pathways among the variables, is deferred to future research.

Suicidal ideation is susceptible to the influence of individual characteristics; it exhibits gender-based variance, and intensifies with declining perceived health status. Although the prevalence of suicidal ideation is significantly higher among female adolescents than their male counterparts, the actual suicide mortality rate is two to four times higher among male individuals (Miranda-Mendizabal et al., 2019). Furthermore, poor subjective health perception has been demonstrated to exhibit a significant positive correlation with suicidal ideation (Nkansah-Amankra et al., 2010). Accordingly, the present study sought to more accurately estimate the relationships among the primary variables—negative parenting attitudes, academic helplessness, OYAP, and suicidal ideation—by controlling for the potential influences of gender and subjective health perception on the dependent variable of suicidal ideation.

**Figure 1:**
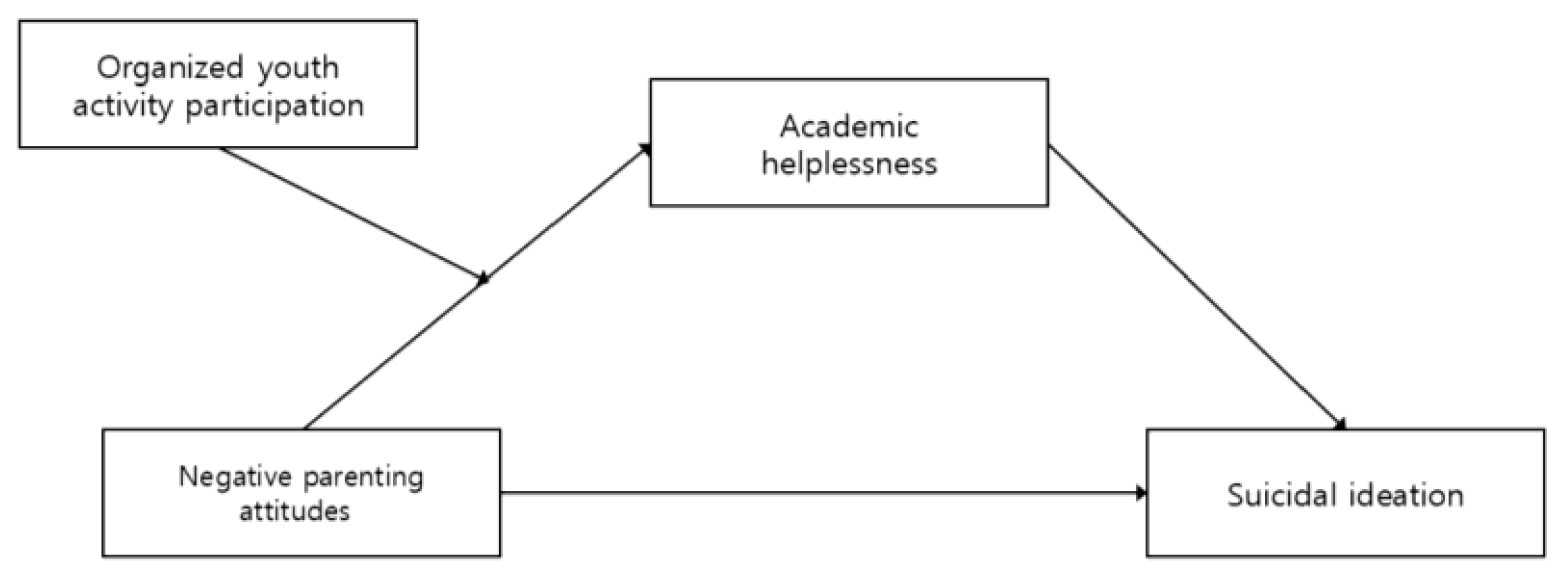
Conceptual diagram of the proposed moderated mediation model. Organized youth activity participation moderates the association between negative parenting attitudes and academic helplessness, which in turn mediates the association between negative parenting attitudes and suicidal ideation.

In summary, adolescents’ perceptions of negative parenting attitudes show a persistent and detrimental association with the psychological and emotional development of adolescent offspring, and are associated with greater academic helplessness and in turn with suicidal ideation. In particular, for Korean adolescents navigating an intensely competitive university entrance environment, academic failure may engender diminished self-worth and self-efficacy, thereby contributing to the emergence of suicidal ideation. From the perspective of behavioral activation theory, organized youth activities may function as a possible effect modifier that buffers this risk pathway by enabling adolescents to autonomously and proactively improve their environments and experience a sense of accomplishment. Accordingly, the present study aimed to examine the association between negative parenting attitudes and adolescent suicidal ideation via the mediation of academic helplessness, and to verify whether the indirect effect on suicidal ideation varies as a function of the level of OYAP, which is proposed to moderate the relationship between negative parenting attitudes and academic helplessness within this pathway (Figure 1).

**H1.**  *Negative parenting attitudes will be positively associated with suicidal ideation*.

**H2.**  *Academic helplessness will mediate the relationship between negative parenting attitudes and suicidal ideation*.

**H3.**  *Organized youth activity participation will moderate the relationship between negative parenting attitudes and academic helplessness, such that the indirect effect on suicidal ideation will be weaker at higher levels of OYAP*.

## 2. Materials and Methods

### 2.1. Participants and Data Collection

This study utilized data from Wave 6 of the Junior High School 1st Grade Panel of the Korean Children and Youth Panel Survey (KCYPS 2018) (National Youth Policy Institute, 2018), provided by the National Youth Policy Institute (NYPI). The KCYPS 2018 was designed to comprehensively examine the growth and developmental trajectories of children and adolescents in South Korea. The sample was recruited using a multi-stage stratified cluster sampling method, targeting students enrolled in the 4th grade of elementary school and the 1st grade of middle school as of 2018. The data employed in this study, collected in 2023, correspond to the period when the panel members were aged 17–18 years. Of the 2590 original panel members available in the 2023 dataset, a total of 366 participants were excluded from the analysis due to missing age information, which precluded determination of their current age. Consequently, a final sample of 2224 participants who provided complete responses was included in the analysis.

### 2.2. Measures

#### 2.2.1. Negative Parenting Attitudes

Negative parenting attitudes were measured using 12 items drawn from the rejection, coercion, and inconsistency subscales of the Korean Adolescent Version of the Parental as Social Context Questionnaire (PSCQ-KA), developed and validated by T. M. Kim and Lee (2017). Participants responded on a 4-point Likert scale (1 = not at all true, 4 = very true), with higher scores reflecting greater perceived negativity in parenting attitudes. Representative items include: “Sometimes I wonder if my parents like me,” “My parents are always telling me what to do,” and “Even when my parents make a promise, I do not know if they will keep it.” In the validation study by T. M. Kim and Lee (2017), the internal consistency reliability (Cronbach’s α) for each subscale was reported as follows: warmth (0.882), rejection (0.797), autonomy support (0.836), coercion (0.780), structure (0.766), and inconsistency (0.752). In the current study, Cronbach’s α for the composite negative parenting attitude scale was 0.864.

#### 2.2.2. Academic Helplessness

Academic helplessness was measured using the Academic Helplessness Scale developed and validated by Bak et al. (2015), which comprises 16 items across four subscales: lack of control beliefs, lack of learning motivation, lack of positive affect, and lack of active engagement. Participants responded on a 4-point Likert scale (1 = not at all true, 4 = very true), with higher scores indicating greater levels of academic helplessness. Representative items include: “I believe I cannot overcome the gap in academic ability on my own,” “I do not want to care about my studies,” and “I do not commit myself to studying even during examination periods.” In the original validation study, Cronbach’s α values for each subscale were as follows: lack of control beliefs (0.911), lack of learning motivation (0.919), lack of positive affect (0.779), and lack of active engagement (0.810). The overall scale demonstrated strong internal consistency in the present study, with a Cronbach’s α of 0.908.

#### 2.2.3. Suicidal Ideation

In the present study, suicidal ideation was assessed using a single item—“I have thoughts of wanting to die”—with no specific reference period specified in the survey instrument, rated on a 4-point Likert scale (1 = not at all true, 4 = very true). This measurement approach has been consistently adopted in prior studies utilizing KCYPS data (Ryu, 2025; Donnelly et al., 2023; Loose et al., 2025), and represents an established method for assessing suicidal ideation in large-scale panel datasets. Examination of the response distribution in Wave 6 revealed that the majority of participants endorsed ‘not at all true’ (1 point; *n* = 1158, 52.1%) or ‘not very true’ (2 points; *n* = 874, 39.3%), whereas 7.8% (*n* = 174) of the participants endorsed “somewhat true” (3 points) and 0.8% (*n* = 18) endorsed “very true” (4 points). Given that approximately 91.4% of responses were concentrated in the lowest two categories, a severe floor effect was evident. Given the skewed distribution of responses and the clinical relevance of distinguishing the presence versus absence of suicidal ideation, the variable was dichotomized such that scores of 3 or above were classified as “suicidal ideation present” and scores of 1 to 2 as “suicidal ideation absent.” Following dichotomization, the prevalence of suicidal ideation was 8.6%.

#### 2.2.4. Organized Youth Activity Participation

Organized youth activity participation (OYAP) is distinguished from unstructured leisure pursuits—such as watching television or spending time with friends—in that it is systematically administered under adult supervision with the explicit aim of enhancing skills and promoting positive development (Mahoney et al., 2006). In South Korea, the domains and types of youth activities are legally defined under the Youth Activity Promotion Act, which seeks to foster the balanced growth of adolescents through participation in diverse activities, including cultural and arts activities, volunteer work, and vocational experience programs (Oh & Ryu, 2017). In the present study, OYAP was measured using a youth activity frequency scale constructed on the basis of this legislative framework. The scale encompasses nine domains: cultural and arts activities, science and information activities, adventure and pioneering activities, career and vocational activities, volunteering activities, international exchange activities, self-development activities, health and medical activities, and environmental preservation activities. For each domain, participants rated the number of times they had participated over the preceding year on a 4-point Likert scale (1 = none, 4 = five or more times). Higher total scores reflect a greater level of engagement in organized youth activities. In the present study, the scale demonstrated satisfactory internal consistency, with a Cronbach’s α of 0.831. Because the nine domains represent qualitatively distinct types of activities, OYAP is conceptualized as a formative participation index rather than a reflective scale. Cronbach’s alpha is reported for transparency but should be interpreted accordingly. Of the total sample, 28.4% of participants reported no participation in any of the nine activity domains, resulting in a positively skewed distribution of OYAP scores.

#### 2.2.5. Control Variables

Gender was treated as a dichotomous variable and dummy-coded as 0 for boys and 1 for girls. Subjective health perception was assessed using a single item asking participants to rate their subjective health status on a 4-point Likert scale ranging from “very unhealthy” to “very healthy”.

### 2.3. Data Analysis

The present study adopted a moderated mediation framework, in which the conditional indirect effect—whereby the magnitude of the mediated pathway varies as a function of the moderator—was simultaneously tested within a single analytical model. The bootstrapping method was employed to evaluate the statistical significance of the indirect effect, given its superior statistical power and relative insensitivity to violations of the normality assumption. All analyses were conducted using IBM SPSS Statistics version 23.0 and the PROCESS Macro for SPSS version 4.2 developed by Hayes (2017), following the sequential steps outlined below.

First, Cronbach’s α coefficients were computed to evaluate the internal consistency of each measure. Second, descriptive statistics—including means, standard deviations (SDs)—were calculated for all variables, and bivariate correlations among the primary variables were examined. Third, PROCESS Macro Model 4 was applied to test the direct effect of negative parenting attitudes on suicidal ideation and the mediating role of academic helplessness, with the significance of the indirect effect evaluated via bootstrapping with 5000 resamples at a 95% confidence interval (CI). Fourth, PROCESS Macro Model 7 proposed by Hayes (2017) was employed to examine whether OYAP moderated the indirect pathway from negative parenting attitudes to suicidal ideation through academic helplessness. To minimize multicollinearity, the independent variable (negative parenting attitudes) and the moderator (OYAP) were mean-centered prior to computing the interaction term in Model 7. Finally, gender and subjective health perception were included as covariates in all models to control for their potential confounding influence on the primary variables.

## 3. Results

### 3.1. Descriptive Statistics

The demographic characteristics of the 2224 participants included in the final analysis are as follows. The sample demonstrated a relatively balanced gender distribution, consisting of 1187 adolescent boys (53.4%) and 1037 adolescent girls (46.6%). The mean age of the participants was 17.99 years (SD = 0.13), and all respondents fell within the age range of 17–18 years.

To ascertain the general characteristics of the measured variables, means and SDs were computed. The descriptive statistics are summarized in Table 1. The mean score for negative parenting attitudes was 23.55 (SD = 5.43) on a scale ranging from 12 to 48; academic helplessness was 31.89 (SD = 7.55) on a scale ranging from 16 to 64; and subjective health perception was 3.14 (SD = 0.52) on a scale ranging from 1 to 4. The dependent variable, suicidal ideation, demonstrated a mean of 0.09 (SD = 0.28), indicating that approximately 8.6% of the respondents reported experiencing suicidal ideation. The mean score for the moderating variable (level of OYAP) was 12.12 (SD = 3.42) on a scale ranging from 9 to 36 and an observed range of 9 to 30 (Table 1).

### 3.2. Correlations

The independent variable, negative parenting attitudes, exhibited significant positive correlations with both the mediating variable, academic helplessness (*r* = 0.473, *p* < 0.01) and the dependent variable, suicidal ideation (*r* = 0.232, *p* < 0.01). This indicates that higher levels of perceived negative parenting attitudes were associated with higher levels of academic helplessness and suicidal ideation. Conversely, negative parenting attitudes showed non-significant negative correlations with gender (*r* = −0.037) and subjective health perception (*r* = −0.038). Academic helplessness demonstrated a significant positive correlation with suicidal ideation (*r* = 0.240, *p* < 0.01). By contrast, it showed significant negative correlations with gender (*r* = −0.102, *p* < 0.01), subjective health perception (*r* = −0.086, *p* < 0.01), and level of OYAP (*r* = −0.062, *p* < 0.01). Finally, the moderating variable, level of OYAP, exhibited significant negative correlations with both academic helplessness (*r* = −0.062, *p* < 0.01) and negative parenting attitudes (*r* = −0.050, *p* < 0.05). This suggests that a higher frequency of participation corresponds with lower levels of academic helplessness and perceived negative parenting attitudes. However, a weak positive correlation was observed between level of OYAP and suicidal ideation (*r* = 0.049, *p* < 0.05), whereas no significant correlations were found between OYAP and either gender or subjective health perception (Table 2).

### 3.3. Mediating Effects

A mediation analysis was conducted using SPSS PROCESS Macro Model 4, with gender and subjective health perception entered as covariates. Given that suicidal ideation, the dependent variable, was dichotomous, logistic regression was applied. The significance of the indirect effect was evaluated on the basis of a 95% CI derived from 5000 bootstrapping resamples; an indirect effect was considered statistically significant at the 0.05 level when the bootstrapped CI did not include zero (Preacher et al., 2007).

First, academic helplessness was significantly predicted by negative parenting attitudes (*B* = 0.6494, *p* < 0.001), such that greater perceived negativity in parenting was associated with higher levels of academic helplessness among adolescents. Second, when both the independent and mediating variables were simultaneously entered into the model to predict suicidal ideation, both negative parenting attitudes (*B* = 0.1108, OR = 1.117, *p* < 0.001) and academic helplessness (*B* = 0.0947, OR = 1.099, *p* < 0.001) were significantly and positively associated with suicidal ideation.

Examination of the bootstrapping results indicated that the indirect effect of negative parenting attitudes on suicidal ideation through academic helplessness was statistically significant (effect = 0.0615, 95% CI [0.0451, 0.0795]), as the CI did not include zero. The direct effect of negative parenting attitudes was also significant (effect = 0.1108, *p* < 0.001), indicating that academic helplessness partially mediated the relationship between these two variables (Table 3).

### 3.4. Moderated Mediation Effects

The SPSS PROCESS Macro (Model 7) proposed by Hayes (2017) was used to determine whether the level of OYAP moderated the relationship between negative parenting attitudes and academic helplessness, and further, to verify the moderated mediation effect on suicidal ideation. Because the dependent variable was dichotomous, a logistic regression-based analysis was conducted, incorporating gender and subjective health perception as control variables. The significance of the indirect effect was evaluated based on a 95% CI generated using 5000 bootstrap resamples (Table 4).

Negative parenting attitudes were significantly and positively associated with academic helplessness (*B* = 0.6425, *p* < 0.001), while OYAP was significantly and negatively associated with academic helplessness (*B* = −0.0863, *p* < 0.05). The interaction between the two variables was also significantly and negatively associated with academic helplessness (*B* = −0.0183, *p* < 0.05), indicating that the association between negative parenting attitudes and academic helplessness varied according to the level of OYAP.

**Figure 2:**
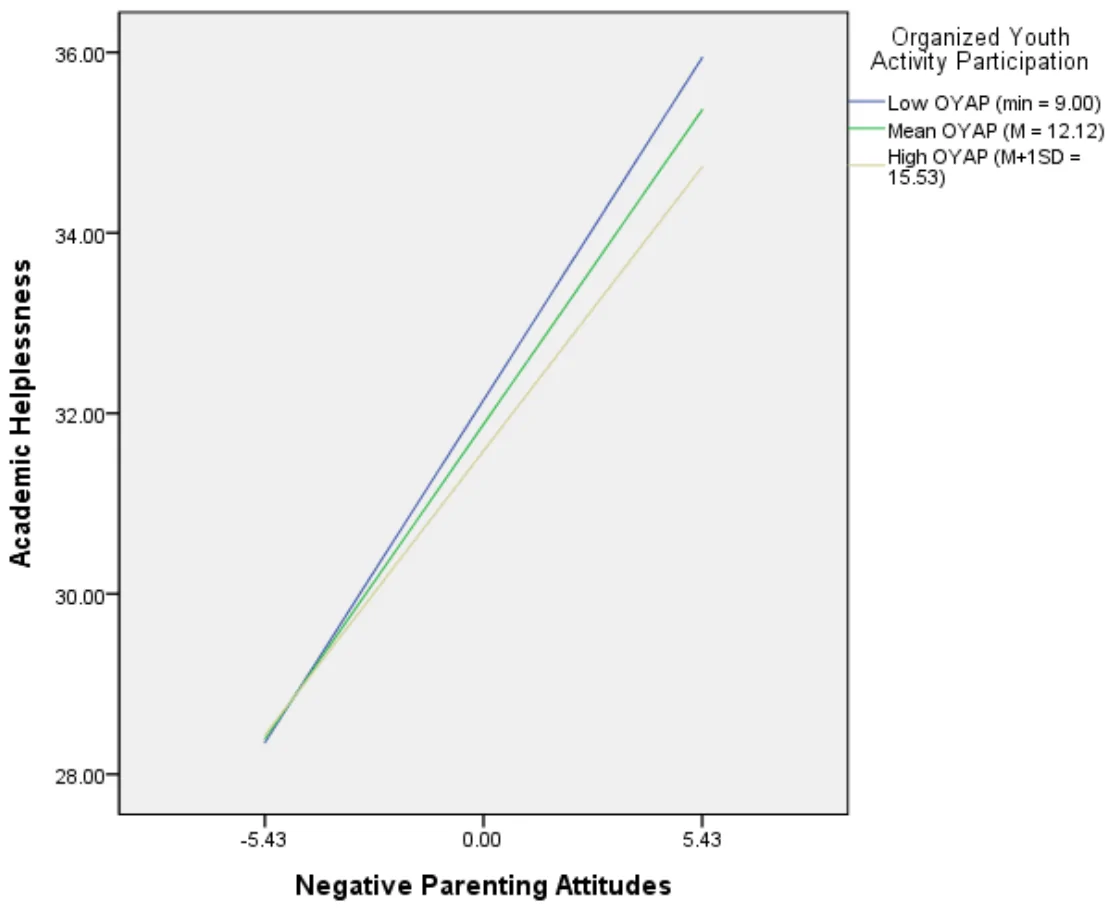
Interaction Plot: Moderating Effect of Organized Youth Activity Participation on the Association Between Negative Parenting Attitudes and Academic Helplessness. Notes. The x-axis represents mean-centered values of negative parenting attitudes (original M = 23.55, SD = 5.43). OYAP values reflect mean-centered scores: Low = −3.12 (observed minimum), Mean = 0, High = +3.42 (mean + 1SD).

To examine the moderating effect more explicitly, a simple slope analysis was conducted at three conditioning values of OYAP: the observed minimum (9.00), the mean (12.12), and one standard deviation above the mean (15.53). The group with a low level of OYAP (9.00, *B* = 0.6995, *p* < 0.001) exhibited a stronger positive association between negative parenting attitudes and academic helplessness than the group with a high level of OYAP (15.53; *B* = 0.5799, *p* < 0.001). In other words, the association between negative parenting attitudes and academic helplessness was most pronounced among adolescents with lower OYAP, and this association attenuated as the level of OYAP increased. A Johnson-Neyman analysis of the a path indicated that there were no statistical significance transition points within the observed range of OYAP, suggesting that the conditional effect of negative parenting attitudes on academic helplessness remained statistically significant across all observed values of OYAP (Figure 2).

The analysis revealed that the indirect effect of negative parenting attitudes on suicidal ideation via academic helplessness varied according to the level of OYAP. The indirect effect was largest at the low level of OYAP (9.00; effect = 0.0662, 95% CI [0.0481, 0.0871]), and gradually decreased at the mean level (12.12; effect = 0.0608, 95% CI [0.0449, 0.0791]) and high level (15.53; effect = 0.0549, 95% CI [0.0403, 0.0723]), although it remained significant across all levels. Furthermore, the index of moderated mediation was significant (index = −0.0017, 95% CI [−0.0037, −0.0001]), confirming that OYAP significantly moderated the indirect pathway from negative parenting attitudes to suicidal ideation via academic helplessness. To assess the robustness of the primary findings, a sensitivity analysis was conducted using an alternative dichotomization threshold (1 = absent, 2–4 = present). The direction and significance of the moderated mediation effect were consistent with the primary analysis (index = −0.1335, 95% CI [−0.2711, −0.0060]), and the conditional indirect effects showed the same pattern across OYAP levels (low: 0.5668 [0.4366, 0.7152]; mean: 0.5205 [0.4015, 0.6494]; high: 0.4699 [0.3529, 0.5992]). These findings suggest that the primary results are robust regardless of the dichotomization threshold applied. Given the positive correlation observed between OYAP and suicidal ideation, a supplementary logistic regression was conducted to examine the direct association between the two variables. Negative parenting attitudes, academic helplessness, gender, and subjective health perception were included as predictors (−2LL = 1087.58, Nagelkerke R^2^ = 0.212). After controlling for all other variables, a significant positive association between OYAP and suicidal ideation was confirmed (B = 0.085, OR = 1.089, 95% CI [1.042, 1.138], *p* < 0.001). This finding is consistent with the positive bivariate correlation between the two variables, and is discussed further in Section 4.

## 4. Discussion

This study investigated the association between negative parenting attitudes and Korean adolescents’ suicidal ideation, and examined the mediating effect of academic helplessness along with the moderated mediation effect of the level of OYAP. The primary findings of this study are discussed below.

First, negative parenting attitudes were significantly and positively associated with adolescent suicidal ideation. This finding aligns with previous research indicating that greater rejection, coercivity, and inconsistent parenting attitudes are associated with increased adolescent suicidal ideation (Macalli et al., 2018; Sachs et al., 2024). These findings suggest that continuous exposure to negative parenting attitudes may be associated with impaired emotion regulation capabilities and amplify psychological distress in adolescents, which may be associated with suicidal ideation in the long term (J. J. Kim & Cho, 2012).

Second, academic helplessness was found to partially mediate the relationship between negative parenting attitudes and suicidal ideation. In other words, negative parenting attitudes were not only directly associated with adolescent suicidal ideation but also associated with greater academic helplessness, which in turn was associated with higher levels of suicidal ideation. These findings align with previous evidence suggesting that more negative parenting attitudes diminish academic motivation and autonomy, predisposing adolescents to poor academic achievement (J. H. Kim, 2022). Furthermore, the accumulation of academic failures can induce a sense of uncontrollability over one’s academic performance, i.e., academic helplessness (Skinner et al., 2016). Particularly during the high school period, characterized by intense competition for university admission, repeated exposure to such experiences is associated with the internalization of learned helplessness—the belief that “effort does not alter the situation.” Consequently, adolescents may become increasingly vulnerable to suicidal ideation (Bano et al., 2019).

Third, the association between negative parenting attitudes and academic helplessness varied according to the level of participation in organized youth activities. Specifically, even when parenting attitudes were perceived as negative, the pathway toward academic helplessness was attenuated among adolescents who maintained high levels of participation. This finding is consistent with prior research suggesting that youth-related or after-school activities function as a possible effect modifier that buffers or offsets the adverse effects of negative parenting (Armstrong & Manion, 2015; Crandall et al., 2024). This also corroborates recent evidence demonstrating that participation in organized youth activities is directly associated with lower levels of academic helplessness (E. T. Lee & Cho, 2025). The moderating effect of OYAP observed in this study can be elucidated through the framework of behavioral activation theory, according to which depression and helplessness stem from a deficit in positive reinforcement and maintenance of avoidance behaviors (Quigley & Dobson, 2017). Adolescents who fail to experience positive reinforcement within the domestic environment because of negative parenting attitudes may exhibit avoidant behaviors in academic settings, thereby becoming susceptible to academic helplessness. However, frequent engagement in organized youth activities provided by schools or local communities increases the frequency of value-driven activities. The consequent repeated experiences of accomplishment and competence can serve as a psychological mechanism consistent with the restoration of self-efficacy and the attenuation of academic helplessness (Joseph et al., 2014; E. T. Lee & Cho, 2025).

Fourth, participation in organized youth activities significantly moderated the pathway from negative parenting attitudes to suicidal ideation mediated by academic helplessness. That is, a moderated mediation effect was confirmed, wherein higher levels of OYAP significantly diminished the magnitude of the indirect effect of negative parenting attitudes on suicidal ideation via academic helplessness. It should be noted, however, that the interaction effect was modest in size (ΔR^2^ = 0.002), and the confidence interval for the index of moderated mediation, although statistically significant, approached zero (index = −0.0017, 95% CI [−0.0037, −0.0001]). These findings suggest that participation in organized youth activities may be associated with lower levels of suicidal ideation by enhancing adolescents’ sense of control, self-efficacy, positive social relationships, and emotional regulation, thereby mitigating the cognitive distortions related to academic failure (Armstrong & Manion, 2015; Mahoney et al., 2006). In other words, the level of OYAP can be interpreted as a possible effect modifier that attenuates the worsening of academic helplessness. In particular, unlike simple leisure activities, organized youth activities—conducted under adult supervision with structured approaches, clear goal-setting, and voluntary participation—are consistent with the behavioral activation theory, which emphasizes regular engagement in meaningful activities as a means of accessing positive reinforcement experiences, thereby potentially reducing avoidance behaviors and increasing positive reinforcement. Indeed, McCauley et al. (2011) demonstrated that the principles of behavioral activation can reduce avoidance behaviors in academic and social contexts. The present findings suggest that OYAP may serve as a context associated with lower levels of depression and suicidal ideation. Additionally, OYAP showed a positive correlation with suicidal ideation, and this positive association remained significant even after controlling for other variables. This finding may reflect selection effects, whereby adolescents experiencing greater psychological distress are more likely to be encouraged to participate in structured youth activity programs. Taken together with the finding that OYAP attenuated the indirect association from negative parenting attitudes to suicidal ideation via academic helplessness, these results suggest that the role of OYAP involves a complex process. Future research should verify the directionality of this association through more precise measurement and longitudinal designs.

The contributions and clinical implications of this study are significant in that the study provides a sophisticated explanation of adolescent suicidal ideation by identifying a model in which environmental factors—specifically, negative parenting attitudes—are associated with suicidal ideation via the cognitive vulnerability of academic helplessness, while demonstrating that the level of participation in organized youth activities moderates this association. These findings suggest that structured strategies that facilitate continuous participation in meaningful and valuable diverse activities within both home and school environments may represent a promising context worthy of further investigation in relation to adolescent suicidal ideation, particularly amid the intense academic competition in South Korea. Particularly during the high school period, when academic achievement is intimately linked to self-worth, OYAP may serve as a valuable resource potentially associated with lower suicidal ideation by providing opportunities for competence, accomplishment, and positive social experiences outside the academic domain. Furthermore, given that adolescents from vulnerable families may face barriers to participation, practical support is necessary to expand community-based organized youth activities and increase the frequency of their engagement.

## 5. Conclusions

In the present study, negative parenting attitudes were found to be significantly associated with suicidal ideation in adolescents, with academic helplessness partially mediating this association. In addition, organized youth activity participation (OYAP) was found to function as a possible effect modifier, attenuating the indirect association between negative parenting attitudes and suicidal ideation through academic helplessness. These findings suggest that OYAP may be a relevant factor worth considering in understanding adolescent suicidal ideation.

However, the present study has several limitations. First, as the sample was drawn from third-year high school students in the KCYPS 2018, the generalizability of the findings to adolescents of different age groups or cultural backgrounds is limited. Furthermore, the use of cross-sectional data precludes the establishment of clear causal relationships among the variables. Second, selection bias may exist due to pre-existing psychological differences between participants based on their level of OYAP. Third, reliance on self-report measures introduces the possibility of common method bias, and the single-item measurement of suicidal ideation carries inherent limitations. In particular, the item used may have captured passive suicidal ideation rather than ideation reflecting specific plans or intent. Although this approach has been widely adopted in prior research (Ryu, 2025; Donnelly et al., 2023; Loose et al., 2025), future studies are encouraged to employ multi-item scales or measures derived from multiple informants. It should also be noted that the items did not specify a uniform reference period, and potential temporal inconsistencies in measurement should be taken into account when interpreting the results. Additionally, as nine types of activities were aggregated into a single composite score, qualitative heterogeneity within the OYAP variable may be present. The inability to fully control for confounding variables such as household socioeconomic status also represents a limitation. Regarding depressive symptoms specifically, the suicidal ideation item was derived from a depression scale; including depressive symptoms as a covariate would therefore risk introducing multicollinearity with the outcome variable, and it was accordingly excluded. With respect to the gender variable, the binary classification employed does not capture the full spectrum of gender identity. Fourth, the absence of multilevel modeling means that clustering effects at the school level were not accounted for, potentially resulting in underestimated standard errors. Future research is encouraged to adopt statistical approaches that reflect hierarchical data structures. Finally, as the model was estimated using logistic regression within the PROCESS macro, the calculation of predicted probabilities, absolute risk differences, and standardized effect sizes was not feasible, which limits the ability to assess the practical magnitude of the interaction effect. Future studies are encouraged to employ statistical software capable of generating these indices and to utilize longitudinal data designs.

## Figures and Tables

**Table 1:** Descriptive Statistics of Study Variables (N = 2224).

Variable	N	Min	Max	M	SD
Gender	2224	0	1	0.47	0.50
Subjective Health	2224	1.00	4.00	3.14	0.52
OYAP	2224	9.00	30.00	12.12	3.42
Negative Parenting	2224	12.00	48.00	23.55	5.43
Academic Helplessness	2224	16.00	64.00	31.89	7.55
Suicidal Ideation	2224	0.00	1.00	0.09	0.28

Notes: Gender: 0 = boys, 1 = girls. Subjective health perception: 1 = very unhealthy, 4 = very healthy. Suicidal ideation: 0 = absent (scores 1–2), 1 = present (scores 3–4). OYAP: theoretical range 9–36, observed range 9–30. Negative parenting attitudes: theoretical range 12–48. Academic helplessness: theoretical range 16–64.

**Table 2:** Correlation Matrix of Study Variables.

Variable	1	2	3	4	5	6
1. Gender	—					
2. Subjective Health	−0.060 **	—				
3. OYAP	0.037	−0.029	—			
4. Negative Parenting	−0.037	−0.038	−0.050 *	—		
5. Academic Helplessness	−0.102 **	−0.086 **	−0.062 **	0.473 **	—	
6. Suicidal Ideation	0.034	−0.107 **	0.049 *	0.232 **	0.240 **	—

Notes: OYAP = Organized Youth Activity Participation. * *p* < 0.05. ** *p* < 0.01.

**Table 3:** Association of Negative Parenting Attitudes with Academic Helplessness (Model 4).

	B	SE	t/Z	*p*	LLCI	ULCI
**Outcome: Academic Helplessness**						
Constant	20.589	1.089	18.911	<0.001	18.453	22.723
Negative Parenting	0.6494	0.026	25.14	<0.001	0.599	0.700
Gender	−1.357	0.281	−4.826	<0.001	−1.909	−0.806
Subjective Health	−1.069	0.270	−3.965	<0.001	−1.598	−0.540
X → M → Y	0.0615	0.009	—	—	0.0451	0.0795
**Model fit**						
R^2^	0.2364					
F(3, 2220)	229.11	*p* < 0.001				

Notes: N= 2224. B = unstandardized coefficient. The outcome suicidal ideation was analyzed using logistic regression (log-odds metric). Bootstrap 95% CI based on 5000 resamples. X = Negative Parenting Attitudes; M = Academic Helplessness; Y = Suicidal Ideation.

**Table 4:** Moderated mediation analysis: Moderating role of OYAP in the indirect effect of negative parenting attitudes on suicidal ideation via academic helplessness (Model 7).

	**B**	**SE**	**t/Z**	***p***	**LLCI**	**ULCI**
**Outcome: Academic Helplessness (a-path)**						
Constant	36.045	0.876	41.148	<0.001	34.327	37.763
Negative Parenting	0.6425	0.026	24.826	<0.001	0.592	0.693
OYAP(W)	−0.0863	0.041	−2.103	0.036	−0.167	−0.006
X × W(interaction)	−0.0183	0.008	−2.443	0.015	−0.033	−0.004
Gender	−1.328	0.281	−4.728	<0.001	−1.879	−0.777
Subjective Health	−1.130	0.270	−4.187	<0.001	−1.659	−0.601
R^2^	0.240					
F(5, 2218)	139.95	*p* < 0.001				
**Variable**	**B**	**SE**	**t/Z**	***p***	**Exp(B)**	**CI**
**Outcome: Suicidal Ideation (logistic, log-odds) ^a^**						
Constant	−3.986	0.667	−5.974	<0.001		
Negative Parenting (X)	0.1108	0.016	7.051	<0.001	1.117	[1.083, 1.152]
Academic Helplessness (M)	0.0947	0.012	7.699	<0.001	1.099	[1.074, 1.126]
Gender	0.4107	0.163	2.524	0.012	1.508	[1.096, 2.075]
Subjective Health	−0.6758	0.153	−4.427	<0.001	0.509	[0.377, 0.686]
**Conditional Indirect Effects of X → M → Y (Bootstrap 95% CI)**						
Low OYAP ^b^ (min = 9.00)	0.0662	0.010	—	—	0.0481	0.0871
Mean OYAP (M = 12.12)	0.0608	0.009	—	—	0.0449	0.0791
High OYAP (M + 1SD= 15.53)	0.0549	0.008	—	—	0.0403	0.0723
**Index of Moderated Mediation**						
OYAP	−0.0017	0.001	—	—	−0.0037	−0.0001
**Model fit: Y model(logistic)**						
−2LL	1101.11					
Modelχ^2^(4)	206.45	<0.001				
Nagelkerke R^2^	0.199					
**Model fit: a-path(OLS)**						
ΔR^2^	0.002					
F(1, 2218)	5.97	*p* = 0.015				

Notes: N = 2224. B = unstandardized coefficient; SE = standard error; LLCI/ULCI = lower/upper limit of confidence interval. The outcome suicidal ideation was analyzed using logistic regression (log-odds metric). Bootstrap 95% CI based on 5000 resamples. X = Negative Parenting Attitudes; M = Academic Helplessness; W = OYAP (Organized Youth Activity Participation); Y = Suicidal Ideation. ^a^ Analyzed using logistic regression (log-odds metric). ^b^ One SD below the mean fell below the minimum observed value of OYAP; therefore, the minimum observed value was used as the low conditioning value.

## Data Availability

The data used in this study are publicly available from the National Youth Policy Institute (NYPI) Korean Children and Youth Panel Survey (KCYPS) 2018. Data can be accessed at https://www.nypi.re.kr.

## Abbreviations

The following abbreviations are used in this manuscript:

KCYPS	Korean Children and Youth Panel Survey
OYAP	Organized Youth Activity Participation
BA	Behavioral Activation
CI	Confidence Interval
